# Impact of Remote Dielectric Sensing on Predicting Worsening Heart Failure During Hospitalization for Heart Failure

**DOI:** 10.3390/jcm13216427

**Published:** 2024-10-26

**Authors:** Teruhiko Imamura, Yu Nomoto, Toshihide Izumida, Nikhil Narang, Koichiro Kinugawa

**Affiliations:** 1 The Second Department of Internal Medicine, University of Toyama, Sugitani 2630, Toyama 930-0194, Japan; 2 Advocate Christ Medical Center, Oak Lawn, IL 60453, USA

**Keywords:** heart failure, hemodynamics, congestion, lung fluid

## Abstract

**Background:** A remote dielectric sensing (ReDS) system quickly quantifies pulmonary congestion. Nonetheless, its efficacy in predicting an in-hospital increase in plasma B-type natriuretic peptide levels, the potential surrogate of worsening heart failure, remains undetermined. **Methods:** Patients who underwent ReDS measurement on admission during their hospitalization in the general wards for heart failure between 2021 and 2022 were eligible. The impact of the baseline ReDS value, completely blinded to the attending clinicians, on the in-hospital increase in plasma B-type natriuretic peptide levels of >100 pg/mL from index admission was evaluated. **Results:** A total of 147 patients admitted with acute-on-chronic heart failure (median age: 79 years; 76 men) were included. The median ReDS value on admission was 28% (25%, 34%). Eighteen patients experienced the primary outcome: plasma B-type natriuretic peptide levels increasing from 461 (207, 790) pg/mL (baseline) to 958 (584, 1290) pg/mL (maximum) (*p* < 0.001). The ReDS value on admission was an independent predictor of the primary outcome, with an adjusted odds ratio of 1.07 (95% confidence interval: 1.01–1.14; *p* = 0.028) with an optimal cutoff of 32%. **Conclusions:** The ReDS system could be a promising tool for predicting in-hospital worsening heart failure in patients hospitalized for heart failure when measured upon admission. The clinical implication of ReDS-guided management of heart failure during index hospitalization requires further studies.

## 1. Background

Worsening heart failure has a deep association with a high degree of morbidity and mortality and is a specific entity distinct from acute-on-chronic heart failure [1]. The majority of data available are derived from patients in the ambulatory care setting. There is a lack of data on specific interventions that improve the outcomes of those hospitalized with acute-on-chronic heart failure and worsening heart failure [2].

Alternate outcomes other than in-hospital mortality are needed, given that the majority of patients, other than those admitted with cardiogenic shock, survive to discharge. Worsening heart failure during inpatient hospitalization is common, and such patients have longer lengths of stay, progression of frailty, and worse clinical outcomes post-discharge [3,4,5]. Identifying potential variables associated with worsening heart failure is needed to both anticipate disease trajectory and offer earlier aggressive interventions that may be favorably disease-modifying [6].

Pulmonary congestion is often the predominant clinical sign in those admitted with acute heart failure [7]. It is often challenging to correctly assess the degree of pulmonary congestion by only utilizing conventional modalities such as chest X-ray. Furthermore, insufficient decongestion and even slight residual congestion are associated with worse clinical outcomes and a higher risk of readmission [8].

**Figure 1:**
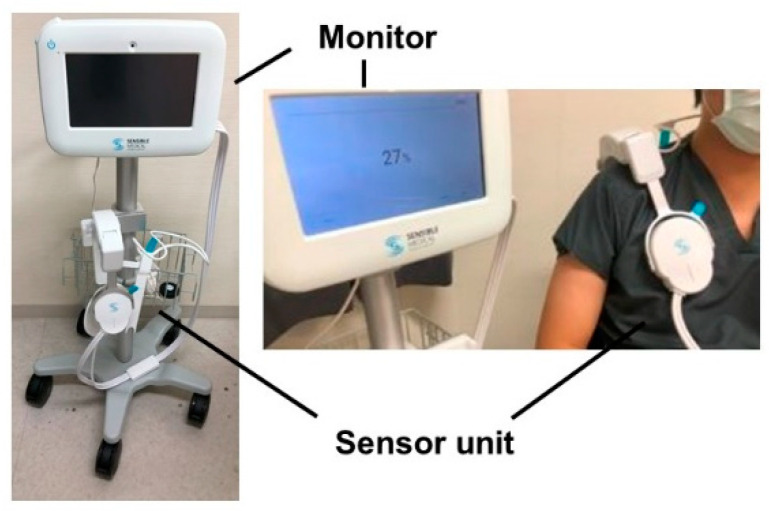
ReDS system.

Remote dielectric sensing (ReDS) systems are a recently introduced non-invasive tool to quantify the degree of pulmonary congestion (Figure 1) [9]. The systems were originally innovated and introduced approximately 20 years ago. Their ability to quantify the amount of pulmonary congestion has been validated by comparing them with other conventional modalities, including chest computed tomography, invasive right heart catheterization, lung ultrasound, and chest X-ray [9]. A ReDS system displays a signal acquisition of the ReDS value, representative of the lung fluid amount as a percentage, within a minute [10]. Due to accumulating evidence, the clinical utility of ReDS systems has been increasing.

Given their ability to accurately quantify pulmonary congestion, we hypothesized that ReDS values on admission might have implications in predicting in-hospital worsening heart failure.

Patients are instructed to sit on the chair. They wear the sensor units for one minute while breathing naturally. The ReDS values, representative of the lung fluid amounts as a percentage of the fluid throughout the whole lung, are displayed on the monitor screen. The lung fluid amount can be estimated non-invasively without any expert technique. The manufacturer’s proposed normal range is between 20% and 35%, although further validations are warranted in real-world clinical practice.

## 2. Methods

### 2.1. Study Design

Patients with heart failure who were hospitalized and underwent ReDS measurement on admission in the general wards were eligible for inclusion. The primary outcome was an in-hospital increase in plasma B-type natriuretic peptide levels of >100 pg/mL from the baseline measurement on admission, a surrogate of worsening heart failure. The prognostic impact of the baseline ReDS values on the primary outcome was evaluated retrospectively. The estimated sample size was calculated as 128 with the following settings: an alpha error of 0.05, a power of 0.85, and an estimated odds ratio of 2.0.

### 2.2. Patient Selection

Patients with heart failure who were hospitalized in the general wards and underwent ReDS measurement on admission in a single large academic center between 2021 and 2022 were eligible for inclusion. Heart failure was diagnosed according to Framingham’s criteria. The detailed inclusion and exclusion criteria are listed in Table 1.

Patients who were hospitalized in high-care units did not receive ReDS measurements and were not included in this study. The presence of pulmonary congestion was obvious in such a cohort, and its degree was assessed by multiple modalities. Patients with inappropriate body sizes for ReDS measurements, such as body mass index below 15.0 or above 30.0, were excluded [10]. Patients with severe lung pathologies were also excluded, given their potential confounding impacts on ReDS values. This included pulmonary pneumonia, lung cancer, and chronic obstructive pulmonary disease. Written informed consent was correctly obtained from participants on admission. The local Institutional Review Board (Ethics Committee, University of Toyama) approved the protocol of the present study (MTK2020007, 3 March 2021).

### 2.3. ReDS Measurement

The ReDS value was measured within one hour of admission prior to the initiation of any therapeutic interventions, in principle, in accordance with standard procedures, as previously detailed. This measurement was conducted in a blinded fashion, unbeknownst to the attending clinicians treating the patients. The ReDS values were evaluated only after the conclusion of the observation period. Thus, the ReDS values did not affect the clinical management at all.

The ReDS system employs non-invasive electromagnetic energy-based technology to quantify the lung fluid volume within a minute. The ReDS values represent the percentage of lung fluid relative to the total lung volume and are displayed on the device monitor (Figure 1) [10]. The ReDS system has received FDA and CE approval for monitoring lung fluid levels.

Specifically, patients were instructed to breathe naturally for 45 s, during which the ReDS values were calculated. The manufacturer-recommended normal range for ReDS values ranges between 20% and 35%, although this range has not been validated in real-world clinical practice [10]. The ReDS values were measured by non-attending clinicians who exclusively served as the research team, ensuring that the values were not referenced in the clinical management.

### 2.4. Measurement of Congestion Score Index of Chest X-Ray

A chest X-ray was also obtained upon admission according to the institutional protocol. The severity of radiographic pulmonary congestion was assessed using a previously reported congestion score index (CSI). The calculation of the CSI was detailed previously in [11].

### 2.5. In-Hospital Management

The attending clinicians treated eligible patients during index hospitalization in a standard manner by guideline-directed medical therapy [12]. The clinicians assessed patients’ congestion by multiple modalities, including physical examination, chest X-ray, and computed tomography, as applicable. As detailed above, the ReDS values were blinded to the attending clinicians.

### 2.6. Clinical Variables

Demographics, comorbidities, laboratory data, echocardiographic data, and medication data were collected on admission as baseline characteristics in addition to the ReDS values. The ReDS value on admission was defined as an independent variable. During index hospitalization, plasma B-type natriuretic peptide levels were measured at least one time per week as per institutional protocol. The primary outcome was defined as an increase in plasma B-type natriuretic peptide levels of >100 pg/mL from baseline values during index hospitalization. The laboratory and medication data at index discharge and length of hospital stay data were obtained as secondary outcomes.

The patients were followed at our institute or affiliated centers after index discharge by board-certified cardiologists at scheduled timings. Death and heart failure readmissions during one year after the index discharge were counted also as secondary outcomes.

### 2.7. Statistical Analysis

Continuous variables were presented as medians (25th and 75th interquartile ranges) and compared between the two groups using the Mann–Whitney U test. Due to the moderate sample size, all continuous variables were treated as non-parametric, regardless of their distributions. Categorical variables were presented as the number of cases (percentage of the total) and compared between the two groups using the chi-square test or Fisher’s exact test. A two-tailed *p*-value of <0.05 was considered statistically significant. Statistical analyses were performed using SPSS Statistics 22 (SPSS Inc, Armonk, IL, USA).

The independent variable was defined as the baseline ReDS value, which was measured on admission blindly by the attending clinicians and assessed retrospectively by the independent researchers. The primary outcome was defined as an increase in plasma B-type natriuretic peptide levels of >100 pg/mL during hospitalization. An increase in plasma B-type natriuretic peptide levels of >200 pg/mL and an increase in plasma B-type natriuretic peptide levels of >30% were also defined as alternative primary outcomes.

Variables that differed significantly between the two groups (an increase in plasma B-type natriuretic peptide levels versus no increase in plasma B-type natriuretic peptide levels) were included in the univariable logistic regression analysis. Potential variables with *p* < 0.05 in the univariable analysis were then included in the multivariable logistic regression analysis to assess the adjusted prognostic impact of the ReDS value.

To evaluate the impact of plasma B-type natriuretic peptide level increases on the one-year composite of death and heart failure readmissions after index discharge, Kaplan–Meier analysis and log-rank tests were conducted.

An ideal cutoff of the ReDS value to predict the primary outcome was calculated by the receiver operating characteristics analysis. The clinical data obtained at index discharge (secondary outcomes) were compared between the two groups stratified by the cutoff of the ReDS value.

## 3. Results

### 3.1. Baseline Characteristics

A total of 147 patients were included. The median age was 79 (71, 83) years, and 76 (52%) patients were men (Table 2). All patients had a primary admission diagnosis of acute or acute-on-chronic heart failure. Of these patients, 21 had coronary artery disease, and 91 had valvular disease. The logarithm of plasma B-type natriuretic peptide levels was 2.4 (2.0, 2.7) pg/mL. The left ventricular ejection fraction was 51% (36%, 65%). With regard to the medications administered, 92 (63%) received loop diuretics, and 46 (31%) received tolvaptan.

### 3.2. Congestion Assessment

**Figure 2:**
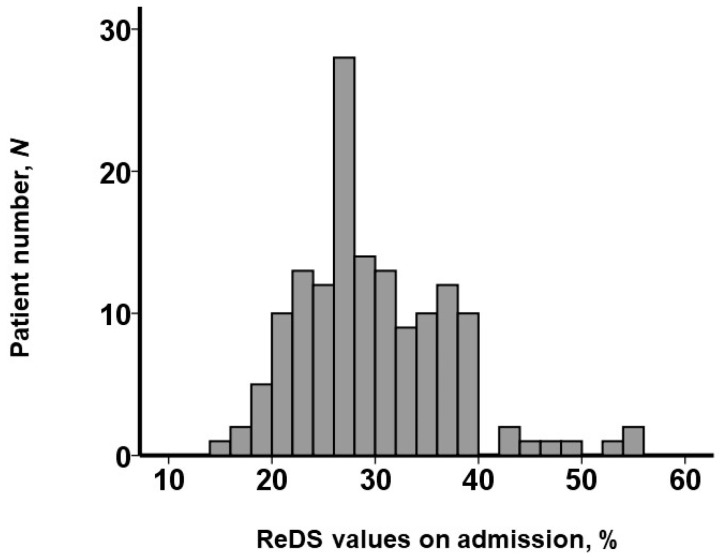
Distribution of ReDS values on admission. ReDS values were distributed widely, with a median value of 28%.

Retrospectively, the ReDS values and CSI were assessed by the independent researchers. The ReDS values on admission were distributed widely, with a median value of 28% (25%, 34%) (Figure 2). The median CSI was 0 (0, 0.7).

### 3.3. Prognostic Impact of ReDS Value on Primary Outcome

**Figure 3:**
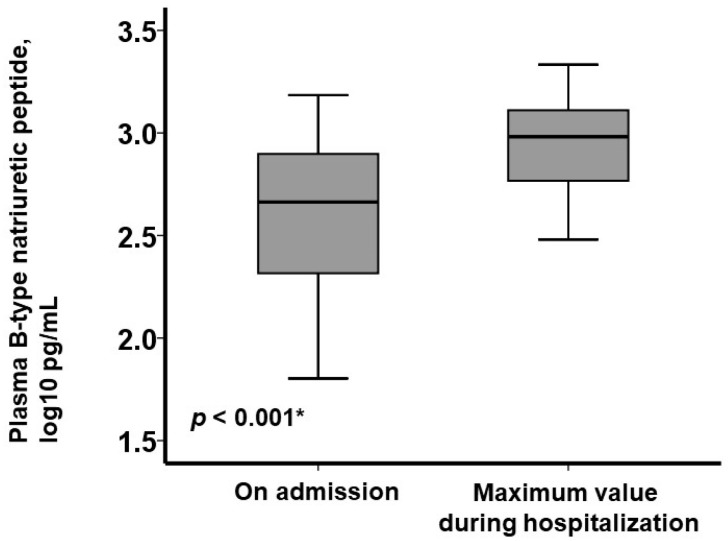
The trend of the logarithm of plasma B-type natriuretic peptide levels on admission and at their maximum among individuals who had worsening heart failure during their index hospitalization, defined as an increase in plasma B-type natriuretic peptide levels of >100 pg/mL (N = 18). * *p* < 0.05 by Wilcoxon’s signed-rank test.

During a median period of 9 (4, 16) inpatient days, no patients died, and 18 patients (12%) experienced an increase in plasma B-type natriuretic peptide levels of >100 pg/mL. Among these 18 patients, plasma B-type natriuretic peptide levels increased significantly from 461 (207, 790) pg/mL (at baseline) to 958 (584, 1290) pg/mL (at maximum) (*p* < 0.001; Figure 3). The dose of diuretics was increased in all of them at the timing of the maximum plasma B-type natriuretic peptide levels.

The baseline characteristics were compared between those with/without an increase in plasma B-type natriuretic peptide levels (Table 2). Patients who experienced the primary outcome had more advanced anemia, hyponatremia, and higher baseline plasma B-type natriuretic peptide levels (*p* < 0.05 for all). The baseline ReDS values were significantly higher in individuals who encountered the primary outcome (*p* = 0.022), whereas the CSI was not significantly different between the two groups (*p* = 0.18). The furosemide equivalent dose on admission was not significantly different between the two groups (20 (0, 30) mg/day versus 20 (10, 30) mg/day (*p* = 0.65)).

**Figure 4:**
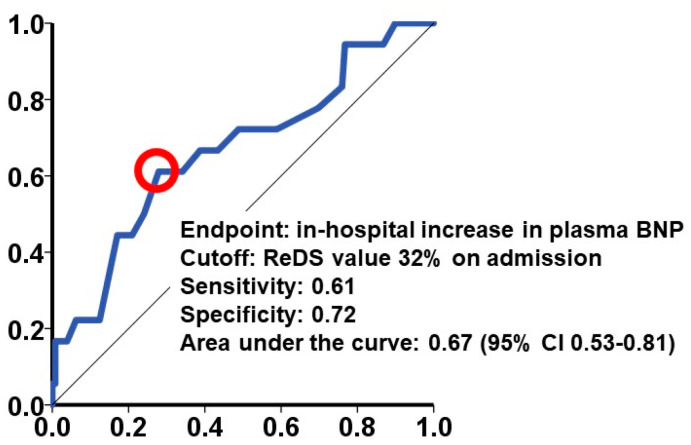
The calculation of the cutoff of the ReDS values on admission for the primary outcome. The cutoff of the ReDS values to predict the primary outcome was calculated as 32%, indicated as a red circle. CI, confidence interval.

Among the four potential variables with *p* < 0.05 in Table 2, higher ReDS values and lower hemoglobin levels remained independently associated with the primary outcome, with adjusted odds ratios of 1.07 (95% confidence interval: 1.01–1.14; *p* = 0.028) and 0.59 (95% confidence interval: 0.42–0.81; *p* = 0.001), respectively (Table 3). The cutoff of the ReDS value to predict the primary outcome was calculated as 32% (sensitivity: 0.61; specificity: 0.72; and area under the curve: 0.67 (Figure 4)).

Other surrogates of worsening heart failure were applied. Thirteen patients had a >200 pg/mL increase in plasma B-type natriuretic peptide levels during index hospitalization. The baseline ReDS value was associated with the endpoint, with an odds ratio of 1.06 (1.02–1.13; *p* = 0.037). Seventeen patients had an over 40% increase in plasma B-type natriuretic peptide levels during index hospitalization. The baseline ReDS value was significantly associated with the endpoint, with an odds ratio of 1.05 (1.01–1.11; *p* = 0.032).

We further evaluated the prognostic impact of the ReDS values on improving heart failure, defined as a decrease in plasma BNP levels of >30% during index hospitalization. The ReDS value was not significantly associated with the endpoint (odds ratio: 0.97; 95% confidence interval: 0.95–1.04; *p* = 0.87).

### 3.4. Secondary Outcome

In total, 47 patients (32%) had ReDS values of >32% at baseline. The clinical data at index discharge were compared between those with/without baseline ReDS values of >32%. The length of hospital stay was significantly longer in individuals with ReDS values of >32% compared with their counterparts (13 days versus 9 days; *p* = 0.026 (Table 4)). The laboratory data at index discharge did not significantly differ according to the ReDS values, except for higher plasma B-type natriuretic peptide levels in individuals with baseline ReDS values of >32% (2.5 (2.1, 2.8) versus 2.2 (1.9, 2.6) pg/mL (*p* = 0.009)). The equivalent dose of furosemide at discharge was significantly higher in patients with ReDS values of >32% compared with their counterparts (60 (40, 80) mg/day versus 30 (20, 40) mg/day (*p* = 0.013)). During index hospitalization, the dose of diuretics was increased in 30 patients (64%) in the high-ReDS group versus 32 patients (32%) in the low-ReDS group (*p* = 0.003).

After index discharge, participants were followed for one year. Eighteen patients encountered a composite of death and heart failure readmissions. Patients who had an increase in plasma B-type natriuretic peptide levels during index hospitalization had a significantly higher cumulative incidence of the composite endpoint (27% versus 14%; *p* = 0.018).

## 4. Discussion

In this retrospective study, we investigated the impact of baseline ReDS values, indicating the degree of pulmonary congestion, on predicting in-hospital increases in plasma B-type natriuretic peptide levels of >100 pg/mL from baseline among individuals who were hospitalized in general wards for heart failure. The baseline ReDS value was independently associated with the primary outcome, with a calculated cutoff of 32% (sensitivity: 0.61, specificity: 0.72, and area under the curve: 0.67). Individuals with baseline ReDS values of >32% had longer in-hospital durations and higher plasma B-type natriuretic peptide levels at index discharge.

### 4.1. Definition of Primary Outcome

The majority of the available literature on worsening clinical status in patients admitted with acute-on-chronic heart failure is pertinent to patients with cardiogenic shock, which has an estimated in-hospital mortality of 25% for heart failure-related causes and 40% mortality for patients with acute myocardial infarction-related cardiogenic shock [13]. On the contrary, there are few studies assessing worsening heart failure by dynamic changes in natriuretic peptides in less-acute patients with heart failure [6,14]. Worsening heart failure in patients without cardiogenic shock is still important to quantify as a clinical endpoint and is associated with longer hospital lengths of stay, progression of frailty, and worse clinical outcomes post-discharge.

On the contrary, an accurate and objective definition of worsening heart failure is challenging. In the present study, we defined an increase in plasma B-type natriuretic peptide levels of >100 pg/mL during index hospitalization as the primary outcome, based on the hypothesis that this primary outcome should be a surrogate of worsening heart failure.

Consistently, patients who satisfied the primary outcome (i.e., an increase in plasma BNP levels) had a higher cumulative incidence of death or heart failure readmission after index discharge. No in-hospital mortality was observed in this cohort, indicating a relatively less sick heart failure cohort who were hospitalized in general wards instead of high-care units.

### 4.2. Prognostic Impact of Baseline ReDS Value

Several risk factors for in-hospital mortality have been proposed [15], whereas there is a scarcity of studies investigating variables associated with in-hospital worsening heart failure. A sub-analysis of the ADHERE study proposed elevated troponin and creatinine levels on admission were associated with in-hospital worsening heart failure, defined as an escalation of medical therapy [6]. In the present study, several factors were associated with in-hospital increases in plasma B-type natriuretic peptide levels in the univariable analysis, including anemia, hyponatremia, and plasma B-type natriuretic peptide levels. However, none of them were significant in the multivariable analysis, except for anemia. Instead, the baseline ReDS value was independently associated with the primary outcome.

The ReDS system is a recently introduced technology to quantify the lung fluid amount non-invasively by utilizing electromagnetic power [10]. ReDS values have a moderate correlation with the percentage of the fluid amount calculated by computed tomography and a mild correlation with other conventional modalities, such as pulmonary artery wedge pressure and lung ultrasound [9]. We should be cautious that the focuses of these conventional modalities are not similar to those of the ReDS system. Most of these conventional modalities represent intravascular congestion, whereas the ReDS value indicates both intravascular and tissue congestion. For example, patients with a low cardiac output due to advanced left ventricular dysfunction often have an elevated pulmonary artery wedge pressure and left ventricular end-diastolic pressure, whereas pulmonary congestion is trivial (i.e., the ReDS value is low). Conversely, the ReDS system is not applicable for detailed differential diagnosis because it gives us data only on the estimated lung fluid volume.

The cutoff of 32% is reasonable considering the previous literature: the equivalent ReDS value to the invasively measured pulmonary artery wedge pressure of 15 mmHg was 28% [16]. As treating clinicians were blinded to the ReDS values, it may have been a challenge to accurately and immediately titrate therapies in response to the clinical exam findings when subclinical congestion was present. Of note, all participants were hospitalized in general wards, and their pulmonary congestion might have been relatively less obvious and challenging to accurately assess without the ReDS system. Such an underestimation of pulmonary congestion and underuse of diuretics during the early hospitalization period might have caused in-hospital worsening heart failure. As a result, the in-hospital stays were longer and the equivalent doses of furosemide at index discharge were eventually higher in patients with high ReDS values at baseline.

Chest X-ray is another practical and essential modality to qualify pulmonary congestion [11]. We do not deny the utility of this conventional modality at all. However, the CSI did not significantly differ between those with/without the primary outcome. Chest X-rays may be too insensitive to detect subclinical congestion for the majority of clinicians; thus, the ReDS system might have an advantage in accurately assessing abnormal lung fluid volumes, particularly when not overtly apparent by traditional modalities.

### 4.3. Clinical Implication

In the present study, the ReDS values were measured blindly by the attending clinicians caring for the study cohort. Based on our findings, ReDS values on admission may be useful for quantifying pulmonary congestion and risk stratifying patients about in-hospital courses beyond conventional modalities such as chest X-rays, particularly when patients are relatively less sick and their pulmonary congestion is not obvious in other conventional modalities. Of note, the accurate assessment of pulmonary congestion by chest X-ray alone is sometimes challenging even for expert clinicians.

Conversely, the clinical utility of ReDS systems might relatively decrease in patients with severe pulmonary congestion admitted to intensive care units. Such patients usually have obvious congestive symptoms, which do not necessarily require specific modalities to quantify pulmonary congestion, including ReDS systems. Such patients usually receive multiple modalities to assess pulmonary congestion, including right-heart catheterization.

In individuals with higher baseline ReDS values, heart failure medications should be considered to be more aggressively up-titrated to mitigate the downstream risk of worsening clinical status and possible readmission.

Several recent studies in the literature have proposed the clinical advantage of ReDS-guided management for outpatient follow-up or at index discharge [17,18], whereas no studies have investigated its implication for in-hospital management. Optimal in-hospital management should lead to successful post-discharge clinical outcomes. Given our findings, ReDS-guided management might further improve in-hospital clinical outcomes, although further large-scale prospective randomized controlled trials are needed to validate this strategy. Given the moderate predictive power of ReDS values, a combination of other clinical indicators and assessment methodologies, such as lung ultrasound and biomarkers, may further enhance the accuracy of prediction [19,20]. Notably, chest X-ray, a conventional and practical tool, could not predict worsening heart failure.

### 4.4. Limitations

This study was not without limitations. Given that ReDS technology has only recently become clinically available, the sample size was small. Both the small sample size and small event numbers limited the statistical power and the statistical strength of the multivariable analysis. A larger-scale study would enable us to conduct a multivariable analysis with various co-predictors of the primary outcome. These co-predictors may be useful for estimating the primary outcome when used together with the ReDS system. While ReDS technology has the potential to non-invasively quantify lung fluid levels while breathing naturally, its validation across diverse clinical scenarios remains limited [21]. For instance, individuals hospitalized in high-care units were excluded, as ReDS values may not be accurately measured in such critical settings (refer to the Methods Section). Clinically evident pulmonary congestion can be assessed using various other modalities, including invasive right-heart catheterization, and may not be the primary target for the ReDS system. Patients with subclinical congestion are likely better candidates for ReDS measurements. The applicability of ReDS systems in various clinical scenarios requires further studies, including a reduced ejection fraction versus a preserved ejection fraction, young versus old, the presence versus absence of significant valvular disease, and lean versus obese. This study serves as a proof-of-concept, and further research involving larger-scale multi-institutional cohorts is warranted to validate these findings and establish the broader applicability of the ReDS technology.

We set the primary outcome as an increase in plasma B-type natriuretic peptide levels of >100 pg/mL, which is probably a surrogate of worsening heart failure. We attempted several other modified endpoints. Given the lack of a gold standard to define worsening heart failure, any definitions might receive some criticism. The strength of our definition is its objectiveness. The limitation of our definition is the lack of any incorporations of subjective parameters, such as heart failure symptoms and signs.

No studies have investigated the cost-effectiveness of ReDS-guided management, including the present study. We should buy or rent the system as an initial cost. Further studies are warranted to demonstrate whether ReDS-guided management may not only improve clinical outcomes versus conventional management but also be cost-effective.

Lastly, we did not follow the trends of the ReDS values during index hospitalization. Baseline characteristics and any therapeutic interventions may affect ReDS values and successive clinical outcomes. The trajectory of ReDS values should give us further insights into predicting the clinical outcomes beyond one-time ReDS value measurements.

## 5. Conclusions

The ReDS system, when measured upon admission, holds promise as a tool for predicting in-hospital worsening heart failure in patients admitted to general wards for heart failure. The use of ReDS values on admission as a basis for aggressive therapeutic interventions to improve clinical outcomes requires further investigation and validation. Future studies should focus on larger cohorts and diverse clinical settings to confirm the efficacy of ReDS-guided strategies and their impact on patient outcomes.

## Figures and Tables

**Table 1:** Inclusion and exclusion criteria.

Inclusion criteria
Diagnosed with heart failure
Hospitalized in general wards
Available ReDS data
No obvious lung diseases
Having appropriate physics for ReDS measurements
Body mass index between 15.0 and 30.0
Able to sit and wear ReDS devices
Aged equal to or above 20 years
Agreed to participate
Exclusion criteria
Disagreed to measure ReDS values
Hospitalized in high-care units
Scheduled invasive intervention
Having obvious lung diseases
Body mass index below 15.0 or above 30.0
Abnormal physics for ReDS measurements
Unable to sit for ReDS measurements

**Table 2:** Baseline characteristics on admission.

	Total (*N* = 147)	BNP Increase (*N* = 18)	No BNP Increase (*N* = 129)	*p*-Value
Demographics				
Age, years	79 (71, 83)	83 (75, 85)	78 (70, 83)	0.053
Men	76 (52%)	7 (39%)	69 (53%)	0.18
Body mass index, kg/m^2^	22.1 (20.1, 25.3)	20.9 (18.6, 23.7)	22.2 (20.2, 26.0)	0.13
Systolic blood pressure, mmHg	117 (98, 133)	112 (97, 127)	118 (98, 133)	0.64
Pulse rate, bpm	75 (69, 85)	72 (68, 83)	75 (69, 85)	0.65
New York Heart Association class I/II/III/IV	0/78/54/15	0/8/7/3	0/70/47/12	0.32
Comorbidity				
Hypertension	100 (68%)	11 (61%)	89 (69%)	0.34
Dyslipidemia	66 (45%)	9 (50%)	57 (44%)	0.41
Diabetes mellitus	49 (33%)	6 (33%)	43 (33%)	0.61
Coronary artery disease	21 (14%)	4 (22%)	17 (13%)	0.3
Valvular disease	91 (62%)	8 (44%)	83 (64%)	0.087
Heart failure	147 (100%)	18 (100%)	129 (100%)	-
Atrial fibrillation	68 (46%)	9 (50%)	59 (46%)	0.46
Chronic kidney disease	109 (74%)	16 (89%)	93 (72%)	0.13
History of stroke	21 (14%)	4 (22%)	17 (13%)	0.3
Laboratory data				
Hemoglobin, g/dL	12.4 (11.1, 13.7)	10.3 (9.6, 12.0)	12.6 (11.4, 13.9)	<0.001 *
Serum creatinine, mg/dL	1.1 (0.8, 1.5)	1.0 (0.8, 1.5)	1.1 (0.8, 1.5)	0.85
Serum sodium, mEq/L	139 (138, 142)	137 (132, 142)	140 (138, 142)	0.032 *
Plasma BNP, log_10_ pg/mL	2.4 (2.0, 2.7)	2.7 (2.3, 2.9)	2.3 (2.0, 2.7)	0.044 *
Echocardiographic data				
Left ventricular end-diastolic diameter, mm	53 (46, 61)	51 (43, 62)	53 (47, 60)	0.69
Left ventricular ejection fraction, %	51 (36, 65)	48 (30, 67)	51 (38, 64)	0.55
Left atrial diameter, mm	44 (37, 52)	50 (36, 63)	43 (38, 52)	0.20
Medications				
Beta-blocker	96 (65%)	10 (56%)	86 (67%)	0.25
Renin–angiotensin system inhibitor	108 (73%)	12 (67%)	96 (74%)	0.49
Angiotensin receptor neprilysin inhibitor	36 (24%)	4 (22%)	32 (25%)	0.81
Mineralocorticoid receptor antagonist	65 (44%)	6 (33%)	59 (46%)	0.23
Sodium–glucose cotransporter 2 inhibitor	52 (35%)	6 (33%)	46 (36%)	0.92
Loop diuretics	92 (63%)	12 (67%)	80 (62%)	0.46
Furosemide equivalent dose, mg/day	20 (10, 30)	20 (0, 30)	20 (10, 30)	0.65
Vasopressin type 2 receptor antagonist	46 (31%)	7 (39%)	39 (30%)	0.31
Intravenous inotropes	12 (8%)	2 (11%)	10 (8%)	0.63
Intravenous vasodilators	14 (10%)	3 (17%)	11 (9%)	0.27
Congestion parameter				
ReDS value, %	28 (25, 34)	34 (26, 36)	27 (25, 33)	0.022 *
Congestion score index	0 (0, 0.7)	0.3 (0, 0.5)	0 (0, 0.5)	0.18

The baseline characteristics obtained on admission were displayed and stratified by the achievement of the primary outcome, which was defined as an increase in plasma B-type natriuretic peptide levels of >100 pg/mL from baseline during index hospitalization. Continuous variables were stated as medians (25% interquartile and 75% interquartile) and compared between the two groups using the Mann–Whitney U test. Categorical variables were stated as numbers and percentages and compared between the two groups using the chi-square test or Fischer’s exact test as appropriate. BNP, B-type natriuretic peptide; ReDS, remote dielectric sensing; * *p* < 0.05.

**Table 3:** Potential variables associated with achieving the primary outcome.

	Univariable Analysis		Multivariable Analysis	
	Odds Ratio (95% CI)	*p*-Value	Odds Ratio (95% CI)	*p*-Value
Hemoglobin, g/dL	0.59 (0.42–0.81)	0.001 *	0.59 (0.42–0.81)	0.001 *
Plasma B-type natriuretic peptide, log_10_ pg/mL	2.66 (0.98–7.21)	0.055		
Serum sodium, mEq/L	0.76 (0.84–1.08)	0.087		
ReDS value, %	1.10 (1.03–1.17)	0.004 *	1.07 (1.01–1.14)	0.028 *

Variables significantly different between the two groups in Table 1 were included in the univariable logistic regression analysis to evaluate their association with the primary outcome. The primary outcome was defined as a plasma B-type natriuretic peptide level increase of >100 pg/mL during index hospitalization. Variables with significance in the univariable analysis were included in the multivariable analysis. ReDS, remote dielectric sensing; CI, confidence interval. * *p* <0.05 by logistic regression analysis.

**Table 4:** Clinical variables at index discharge stratified by the baseline ReDS values.

	ReDS Value > 32% (*N* = 47)	ReDS Value ≤ 32% (*N* = 100)	*p*-Value
Length of hospital stay, days	13 (7, 21)	9 (4, 15)	0.026 *
Systolic blood pressure, mmHg	102 (89, 116)	104 (90, 120)	0.39
Pulse rate, bpm	69 (63, 75)	71 (63, 81)	0.22
Hemoglobin, g/dL	11.8 (10.5, 12.9)	12.1 (10.8, 13.7)	0.20
Serum creatinine, mg/dL	1.1 (0.9, 1.7)	1.1 (0.9, 1.5)	0.51
Serum sodium, mEq/L	138 (137, 140)	139 (137, 141)	0.30
Plasma B-type natriuretic peptide, log_10_ pg/mL	2.5 (2.1, 2.8)	2.2 (1.9, 2.6)	0.009 *
Beta-blockers	38 (81%)	80 (80%)	0.55
Renin–angiotensin system inhibitors	43 (91%)	92 (92%)	0.57
Angiotensin receptor neprilysin inhibitor	14 (30%)	32 (32%)	0.79
Mineralocorticoid receptor antagonists	25 (53%)	61 (61%)	0.24
Sodium–glucose cotransporter 2 inhibitors	23 (49%)	43 (43%)	0.31
Loop diuretics	34 (72%)	67 (67%)	0.33
Furosemide equivalent dose, mg/day	60 (40, 80)	30 (20, 40)	0.013 *
Vasopressin type 2 receptor antagonist	20 (43%)	41 (41%)	0.50

The clinical variables obtained at index discharge (secondary outcomes) were stratified by the cutoff of the ReDS value. The cutoff was calculated to estimate the occurrence of primary outcome by receiver operating characteristics analysis. Continuous variables were stated as medians (25% interquartile and 75% interquartile) and compared between the two groups using the Mann–Whitney U test. Categorical variables were stated as numbers and percentages and compared between the two groups using the chi-square test or Fischer’s exact test as appropriate. ReDS, remote dielectric sensing; * *p* < 0.05.

## Data Availability

The original contributions presented in the study are included in the article, further inquiries can be directed to the corresponding author/s.
